# Effect of Wearable Sensor-Based Exercise on Musculoskeletal Disorders in Individuals With Neurodegenerative Diseases: A Systematic Review and Meta-Analysis

**DOI:** 10.3389/fnagi.2022.934844

**Published:** 2022-07-26

**Authors:** Xin Li, Zhengquan Chen, Yiming Yue, Xuan Zhou, Shuangyu Gu, Jing Tao, Haibin Guo, Meiwen Zhu, Qing Du

**Affiliations:** ^1^Department of Rehabilitation, Xinhua Hospital, Shanghai Jiao Tong University School of Medicine, Shanghai, China; ^2^Institute of Rehabilitation Engineering and Technology, University of Shanghai for Science and Technology, Shanghai, China; ^3^Chongming Branch of Xinhua Hospital, School of Medicine, Shanghai Jiao Tong University, Shanghai, China

**Keywords:** neurodegenerative diseases, Parkinson’s disease, Alzheimer’s disease, wearable sensor-based exercise, musculoskeletal disorders, balance, dynamic postural control

## Abstract

**Background:**

The application of wearable sensor technology in an exercise intervention provides a new method for the standardization and accuracy of intervention. Considering that the deterioration of musculoskeletal conditions is of serious concern in patients with neurodegenerative diseases, it is worthwhile to clarify the effect of wearable sensor-based exercise on musculoskeletal disorders in such patients compared with traditional exercise.

**Methods:**

Five health science-related databases, including PubMed, Cochrane Library, Embase, Web of Science, and Ebsco Cumulative Index to Nursing and Allied Health, were systematically searched. The protocol number of the study is PROSPERO CRD42022319763. Randomized controlled trials (RCTs) that were published up to March 2022 and written in English were included. Balance was the primary outcome measure, comprising questionnaires on postural stability and computerized dynamic posturography. The secondary outcome measures are motor symptoms, mobility ability, functional gait abilities, fall-associated self-efficacy, and adverse events. Stata version 16.0 was used for statistical analysis, and the weighted mean difference (WMD) was selected as the effect size with a 95% confidence interval (CI).

**Results:**

Fifteen RCTs involving 488 participants with mean ages ranging from 58.6 to 81.6 years were included in this review, with 14 of them being pooled in a quantitative meta-analysis. Only five included studies showed a low risk of bias. The Berg balance scale (BBS) was used in nine studies, and the pooled data showed a significant improvement in the wearable sensor-based exercise group compared with the traditional exercise group after 3–12-week intervention (WMD = 1.43; 95% CI, 0.50 to 2.36, *P* = 0.003). A significant change in visual score was found both post-assessment and at 1-month follow-up assessment (WMD = 4.38; 95% CI, 1.69 to 7.07, *P* = 0.001; I2 = 0.0%). However, no significant differences were found between the two groups in the secondary outcome measures (all *p* > 0.05). No major adverse events were reported.

**Conclusion:**

The wearable sensor-based exercise had advantages in improving balance in patients with neurodegenerative diseases, while there was a lack of evidence in motor symptoms, mobility, and functional gait ability enhancement. Future studies are recommended to construct a comprehensive rehabilitation treatment system for the improvement in both postural control and quality of life.

**Systematic Review Registration:**

http://www.crd.york.ac.uk/prospero/, identifier CRD42022319763.

## Introduction

Neurodegenerative diseases (e.g., Alzheimer’s disease, Parkinson’s disease, motor neuron diseases, or amyotrophic lateral sclerosis) are caused by the progressive degeneration of neurons and/or their myelin sheaths and mainly characterized by the deposition of proteins showing altered physicochemical properties in the brain and in peripheral organs (Dugger and Dickson, 2017; Kovacs, 2017), such as degeneration and death of dopaminergic neurons in substantia nigra, or tangle of intracellular neurofibrillary. Symptoms of neurodegenerative diseases vary depending on the mechanism of degeneration and the corresponding brain region (Erkkinen et al., 2018). As degeneration increases, patients may develop different types of dysfunctions, including cognitive or motor impairments (Pender et al., 2020; Aarsland et al., 2021). Moreover, neurodegenerative diseases are among the most serious health problems affecting the life expectancy of millions of people worldwide (Kingwell, 2019; Dommershuijsen et al., 2020), with an estimated incidence rate from approximately 17 per 100,000 to 11.08 per 1000 person-years in Parkinson’s disease (PD) (Hirsch et al., 2016) and Alzheimer’s disease (AD) (Niu et al., 2017), respectively.

Previous study suggested that the main motor symptom, which was associated with neurodegeneration, increases gradually as the disease progresses (Ray and Agarwal, 2020), is the postural control abnormalities because the degeneration of the nervous system influences the integration of sensory information, the formation of motor patterns (which can be understood as the central nervous system generating the imagination of the movement), and the disorder of muscle control (Ryan et al., 2014). A growing body of research demonstrated that patients with neurodegenerative diseases may present with decreased balance, abnormal gait (Morel et al., 2020), a higher tendency to fall (Schell et al., 2019), and frailty (Swanson and Robinson, 2020; Waite et al., 2021), which brings about higher morbidity and mortality and also turns into significant healthcare concerns.

Cass et al. proposed that the ability to control postural balance is essential to perform most of the daily life activities, allowing people to maintain an active lifestyle, and avoiding falls (Cass, 2017). Motor intervention is a critical pathway for improving both balance and postural control in people with neurodegenerative diseases. The traditional exercise protocol consists of repeated balance training and gait relearning under the guidance of the therapist (Ni et al., 2018; Meng et al., 2020; Okada et al., 2021), while wearable systems are a promising solution to provide quantitative and meaningful clinical information about progress in a rehabilitation pathway, with personalized biofeedback or tele-therapy that can be administered in the comfort of settings for those with progressive neurological conditions (Porciuncula et al., 2018). Well-documented evidence suggested that the involvement of wearable sensor-based exercise, which means a cueing rehabilitation based on devices providing visual, auditory, or vibrotactile biofeedback (Carpinella et al., 2017), can provide patients with instant and sensitive biofeedback about the user’s performance and build an interactive environment for supporting motor learning (Argent et al., 2019). The sensors can accurately measure body motion and capture the tendency of incorrect action patterns to promote the learning of postural control (Carpinella et al., 2017; Jakob et al., 2021; Meng et al., 2021). Studies have supported that a wearable sensor-based intervention model is well suited to impact a movement disorder in people with mild cognitive impairment (Schwenk et al., 2016), as they can serve as a “sixth sense,” promote the central organization of multiple sensory inputs through external feedback (Gomez-Pinilla and Hillman, 2013; Gera et al., 2018), guide the formation of movement patterns in the brain (internal feedback) to control posture (Donath et al., 2016; Shih et al., 2016; Conradsson et al., 2017; Silva et al., 2017), and give a motivating effect due to game-based features (Horak et al., 2015).

Overview articles have discussed the potential of wearable sensor-based exercise for improving clinically relevant motor performances, such as postural stability or gait, which are important for safe ambulation and mobility-related quality of life (Horak et al., 2015), and a previous meta-analysis, including eight randomized controlled trials (RCTs), provided evidence for a positive effect of wearable sensor-based exercise on static steady-state balance of healthy and various patient populations in studies with usual care controls and studies with conventional balance training controls (Gordt et al., 2018). However, to our knowledge, it remains unclear whether the wearable sensor-based exercise can effectively improve musculoskeletal disorders in individuals with neurodegenerative diseases. Therefore, the purpose of this study was to clarify the effectiveness of wearable sensor-based exercise for musculoskeletal concerns in patients with neurodegenerative diseases.

## Methods

The Preferred Reporting Item for Systematic Reviews and Meta-Analyses (PRISMA) guidelines were used to structure our review. PROSPERO was used to enroll the protocol for this meta-analysis (No. CRD42022319763).

### Search Strategy

Two reviewers (Xin Li and Zhengquan Chen) independently conducted an extensive search in five health science databases, including PubMed, Cochrane Library, Embase, Web of Science, and Ebsco Cumulative Index to Nursing and Allied Health, up to March 2022. The following search terms and their synonyms: “neurodegenerative diseases,” “Parkinson’s disease,” “Alzheimer’s disease,” “motor neuron diseases,” “amyotrophic lateral sclerosis,” “biofeedback,” “sensor,” “exercise,” and “postural control” (Table 1), were used. The search strategies are given in Supplementary Appendix 1.

**TABLE 1:** Terms used in the search strategy.

Search strategy
#1 Neurodegenerati* OR Parkinson* OR PD OR Alzheimer* OR “Amyotrophic Lateral Sclerosis” OR ALS OR “Motor Neuron Diseases”
#2 Biofeedback OR Sensor* OR Inertial OR IMU OR Acceleromet* OR Actigraph* OR Gyroscope* OR Magnetometer* OR “Virtual Reality” OR Exergam*
#3 Exercise* OR Physical Activity OR Sport* OR Training
#4 Balance OR Postur* OR “Motor Control” OR Gait OR Propriocepti*
#5 random* OR allocation OR placebo OR “single-blind” OR “double-blind” OR RCT
#6 #1 AND #2 AND #3 AND #4 AND #5

* Is used as a wildcard for truncated word retrieval.

We also searched the reference lists of the included studies and reviews of similar topics to identify additional eligible studies. This systematic review only included RCTs written in English. If there was a disagreement, the full text of the article was checked and discussed, if necessary, with third-party adjudication (Meiwen Zhu).

### Eligibility Criteria

The following criteria that followed PICOS strategy were used to determine whether studies were eligible: (1) population: patients who were diagnosed with neurodegenerative diseases, including Parkinson’s disease, Alzheimer’s disease, motor neuron diseases, and amyotrophic lateral sclerosis; (2) intervention: wearable sensor-based exercise, including self-developed sensors or commercial sensors, which can provide visual, auditory, or vibrotactile biofeedback; (3) comparisons: therapeutic exercise without the use of sensors; (4) outcome measures: primary outcome being balance and secondary outcomes being motor symptoms, mobility ability, functional gait abilities, and fall-associated self-efficacy; and (5) study design: RCTs.

### Study Selection

Two reviewers (Yiming Yue and Shuangyu Gu) independently screened the studies using the eligibility criteria. The titles and abstracts were initially screened, and then, the full texts of the remaining articles were extensively reviewed. Disagreements were resolved by discussion and rechecking the articles.

### Outcome Measurements and Data Extraction

Considering the progression of neurodegenerative diseases, changes in the outcomes were compared between the wearable sensor-based exercise and controls. A change in balance was the primary outcome for extraction. Balance was assessed by evaluated by the Berg balance scale (BBS) or sensory organization test (SOT). A change in motor symptoms evaluated by the Unified Parkinson’s Disease Rating Scale-III (UPDRS-III), mobility ability evaluated by the timed “Up and Go” test (TUG), 10-meter walking test (10MWT), and 39-item Parkinson’s Disease Questionnaire (PDQ-39), functional gait abilities evaluated by the dynamic gait index (DGI), and fall-associated self-efficacy evaluated by the activities-specific balance confidence (ABC) scale were also extracted as the secondary outcomes.

Two reviewers (Qing Du and Haibin Guo) were paired up to retrieve the information and data from the included RCTs. Disagreements were resolved by discussion with a third reviewer (Xin Li). The following data were extracted: first author, published year, country, the number, gender, and age of participants; the duration, severity, and medication of the neurodegenerative diseases; sensor type; outcome measures; time points; and dropout rate. The details of the intervention methods (frequency, intensity, time, and type of exercise) in both the control group and sensor-based intervention group were also extracted. Adverse events were also collected to determine the safety of the wearable sensor-based exercise protocols.

Mean, standard deviation (SD), and the sample size were extracted for the outcome measures in each group (i.e., active and sham) for the pooled analysis. Published protocols were referenced and the corresponding authors were contacted for additional data when data were not directly available from the article.

### Risk of Bias Assessment

The two reviewers (Xuan Zhou and Jing Tao) independently used the Cochrane risk of bias assessment tool (RoB 2.0) for RCTs to assess the methodological quality of the included studies. RoB 2.0 provides five domains and gives the overall risk of bias evaluation at the end. The five domains in RoB 2.0 are (1) the randomization process, (2) deviations from the intended interventions, (3) missing outcome data, (4) measurement of the outcome, and (5) selection of the reported result. For missing outcome data in individual studies, we stipulated a low risk of bias for loss to follow-up of less than 10% and a difference of less than 5% in missing data between intervention and control groups. Publication bias was assessed through visual inspection of funnel plots for each outcome in which 10 or more eligible studies were identified.

### Meta-Analysis and Subgroup Analyses

We used Stata version 16.0 (Stata Corp., College Station, TX, United States) to conduct this meta-analysis. The weighted mean difference (WMD) with 95 percent confidence intervals (Cls) was calculated to represent the effect size. The *I*^2^ test was used to estimate the heterogeneity. A random-effects model was adopted when there was a significant heterogeneity (*I^2^* > 50%); otherwise, a fixed-effects model was used. Egger’s test was used to identify the publication bias of the main outcome measures. The *p*-value at the 0.05 level was considered statistically significant. Moreover, a sub-group analysis was performed on outcome measures of different follow-up time points according to the included studies.

### Quality of Evidence

The Grading of Recommendations Assessment, Development, and Evaluation (GRADE) approach was conducted in this systematic review and meta-analysis to determine the quality of the evidence provided by RCTs. Detailed GRADE guidance was used to assess the overall risk of bias, imprecision, inconsistency, indirectness, and publication bias and to summarize the results. We categorized each piece of evidence as high, medium, low, or very low quality (Atkins et al., 2004).

## Results

### Identification of Studies

**FIGURE 1:**
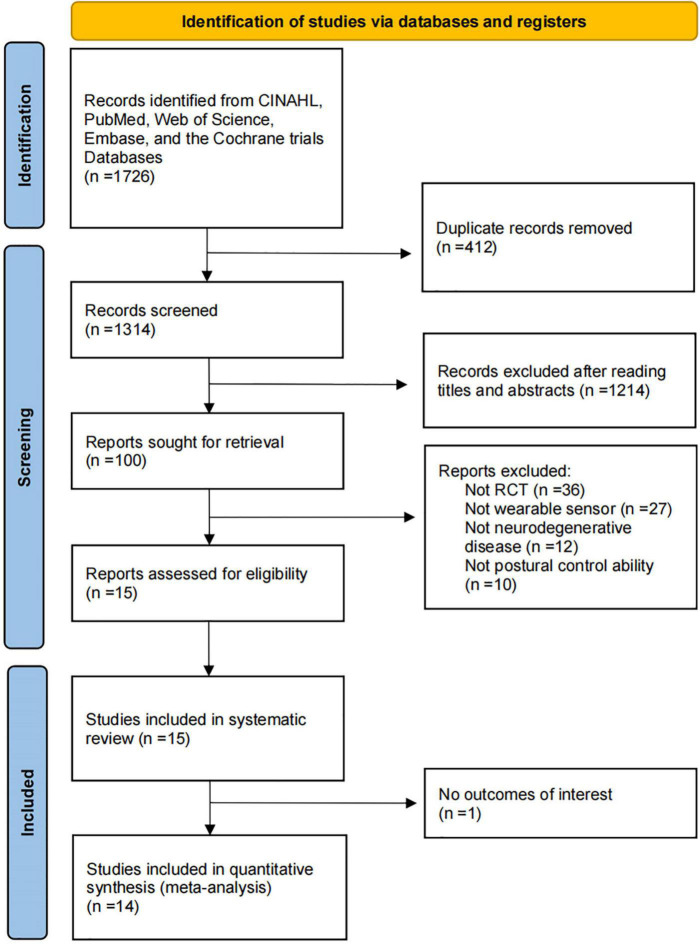
Flowchart of the process of literature search and extraction of studies meeting the inclusion criteria.

We found 1726 relevant articles from five health science databases. After removing duplicates, 1314 article titles and abstracts were screened for relevance. Eventually, 1299 articles were excluded due to not meeting the inclusion criteria or satisfying the exclusion criteria, and 15 articles were included. All these studies were randomized clinical trials. The flowchart of the study selection process is shown in Figure 1.

### Participant and Study Characteristics

The demographic variables, type of sensors, time point, outcome measures, and dropout rate are given in Table 2. The frequency, intensity, time, and type of the exercise protocols in the intervention group and control group are presented in Table 3. A total of 15 articles involved 488 participants with mean ages ranging from 58.6 to 81.6 years, with 14 of them being pooled in a quantitative meta-analysis. The selected RCTs were performed in patients with PD (*n* = 13) (Yen et al., 2011; Nanhoe-Mahabier et al., 2012; Pompeu et al., 2012; van den Heuvel et al., 2014; Liao et al., 2015a,b; Yang et al., 2016; Carpinella et al., 2017; Gandolfi et al., 2017; Ribas et al., 2017; Cikajlo and Potisk, 2019; Santos et al., 2019; Kafle and Rizvi, 2021) and AD (*n* = 2) (Padala et al., 2012; Ugur and Sertel, 2020). Most studies included patients with mild-to-moderate Parkinson’s disease (Hoehn and Yahr stages I to III).

**TABLE 2:** Characteristics of the included studies.

	Author, year	Country	No. of participants (% men)	Age (y), Range/ Mean (SD)	Disease severity (mean ± SD)	Diagnosis	Disease duration (year) mean (SD)	Drug usage	Sensor type	Outcome measures	Time points	Dropout rate
1	Carpinella et al., 2017	Italy	T:37 A:17 (82.3%) C:20 (45.0%)	A: 73.0 (7.1) C:75.6 (8.2)	Hoehn and Yahr stages II to III UPDRS-III (0–56): A:16.6 (6.8) C:22.3 (7.3)	Parkinson’s Disease	A:7.5 (3.2) C:10.3 (5.7)	−	Inertial sensors (TMA); 3D accelerometer, gyroscope, and magnetometer	BBS; 10MWT; UPDRS-III; TUG; ABC; FOGQ; PDQ-39; cop ML sway; cop AP sway	Baseline Intervention: 6–7 weeks Follow-up: 1-month	Post- treatment: 0% End of follow-up: 13.5%
2	Cikajlo and Potisk, 2019	Slovenia	T:20 (45%) A:10 (50%) C:10 (40%)	A:67.6 (7.6) C: 71.3 (8.4)	Hoehn and Yahr stages II to III	Parkinson’s Disease	A:7.1 C:7.1	Dopamine	3D VR Oculus Rift CV1 head-mounted device	BBT; UPDRS; tfftte, atom, IB, tnot, ATI, atips	Baseline Intervention: 3 weeks	Post- treatment: 0% End of follow-up: 0%
3	Gandolfi et al., 2017	Italy	T:70 A:38 (60.5%) C:38 (73.6%)	A:67.45 (7.18) C: 69.84 (9.41)	Modified Hoehn and Yahr (H&Y) stages 2.5 to 3 UPDRS score: A:44.13 (24.05) C:50.76 (24.12)	Parkinson’s Disease	A:6.16 (3.81) C:7.47 (3.90)	−	TeleWii balance training + VR telerehabilitation	BBS; falls; ABC; 10MWT; DGI; PDQ-8	Baseline Intervention: 7 weeks Follow-up: 1 month	Post- treatment: 7.9% End of follow-up: 7.9%
4	Kafle and Rizvi, 2021	India	T:60 A:30 (50.0%) C:30 (43.3%)	A:72.17 (8.19) C:72.40 (6.71)	Hoehn and Yahr stages I to II UPDRS A:30.90 (8.33) C:31.27 (7.78)	Parkinson’s Disease	−	Levodopa or its synergists	Nintendo Wii gaming console	BBS;UST;UPDRS	Baseline Intervention: 7 weeks	−
5	Liao et al., 2015a *	China	T:36 A:12 (50%) C:12 (50%)	A:67.3 (7.1) C: 65.1 (6.7)	Hoehn and Yahr stages I to III A: 2.0 ± 0.7 C:2.0 ± 0.8	Parkinson’s Disease	A:7.9 (2.7) C:6.9 (2.8)	−	VR-based Wii Fit exercise	TUG, PDQ-39, and FES-I	Baseline Intervention: 6 weeks Follow-up: 1 month	Post- treatment: 0% End of follow-up: 2.8%
6	Liao et al., 2015b *	China	T:36 A:12 (50%) C:12 (50%)	A:67.3 (7.1) C: 65.1 (6.7)	Hoehn and Yahr stages I to III A: 2.0 ± 0.7 C:2.0 ± 0.8	Parkinson’s Disease	A:7.9 (2.7) C:6.9 (2.8)	−	VR-based Wii Fit exercise	Gait: Level walking velocity; Stride length; FGA; Sensory organization test	Baseline Intervention 6 weeks Follow-up: 1 month	Post- treatment: 0% End of follow-up: 2.8%
7	Padala et al., 2012	United States	T:22 (27%) A:11 (27%) C:11 (27%)	A:79.3 (9.8) C: 81.6 (5.2)	−	Alzheimer’s Dementia	−	−	Nintendo Wii Fit console	BBS; TT;TUG; ADL; IADL; QOL-AD	Baseline Intervention: 8 weeks	−
8	Pompeu et al., 2012	Brazil	T:32 (53.1%) A:16 C:16	T:67.4 (8.1)	Hoehn and Yahr stages I to II UPDRS: T:9.5 (3.4) A:10.1 (3.8) C:8.9 (2.9)	Parkinson’s Disease	−	Levodopa or its synergists	Nintendo Wii Fit console	UPDRS II; BBS	Baseline Intervention7 weeks Follow-up: 60 days	−
9	Ribas et al., 2017	Brazil	T:20 (60%) A:10 (40%) C:10 (40%)	T:61 (9.11) A:61.7 (6.83) C:60.2 (11.29)	UPDRS: A: 22.5 (11.5–32) C:20.5 (13.5–27.5)	Parkinson’s Disease	A:6.5 (4) C:7 (2.79)	Dopaminergic medication	Nintendo Wii Fit console	BBS; 6MWT Quality-of-life scores: PDQ-39 Mobility	Baseline Intervention: 12 weeks Follow-up: 60 days	Post- treatment: 0% End of follow-up: 0%
10	Santos et al., 2019	Brazil	T:41 A:13 (84.6%) C:14 (78.6%)	A: 61.7 (7.3) C: 64.5 (9.8)	Hoehn and Yahr stages I to III A:1.4 (0.6) C: 1.3 (0.3)	Parkinson’s Disease	A:7.0 (2.8) C:6.5 (2.0)	Levodopa	VR-based Wii Fit exercise	BBS; DGI; TUG Quality-of-life score (PDQ-39): Total	Baseline Intervention8 weeks Follow-up: 2 months	Post- treatment: 0% End of follow-up: 0%
11	Ugur and Sertel, 2020	Turkey	T:32 A:16 (68%) T:16 (75%)	A:73.75 (5.16) C:73.13 (3.54)	−	Alzheimer’s Dementia	−	−	VR-based Wii Fit exercise	Tinetti Gait and Balance Test (Balance); five-time Sit-to-Stand Test; Gait Speed	Baseline Intervention: 6 weeks	Post- treatment: 0% End of follow-up: 0%
12	van den Heuvel et al., 2014	Netherlands	T:33 (60.6%) A:17 (70.6%) C:16 (50%)	A:66.3 (6.39) C:68.8 (9.68)	Hoehn and Yahr stages II and III UPDRS (Total): A:46.0 (19.81) C:52.0 (21.11)	Parkinson’s Disease	−	A: One patient received intestinal levodopa infusion. One patient received an acetylcholine sterase inhibitor	Inertial sensors (Xsens, Enschede, Netherlands)	FRT; BBS; Single-leg test;10MWT; UPDRS; FES PDQ-39 mobility	Baseline Intervention 6 weeks Follow-up: 12 weeks	Post- treatment: 3.0% End of follow-up: 6.1%
13	Yang et al., 2016	China	T:23 (60.9%) A:11 (63.6%) C:12 (58.3%)	A:72.5 (8.4) C:75.4 (6.3)	Hoehn and Yahr stages II and III UPDRS: A:22.5 (12.1) C:21.7 (14.4)	Parkinson’s Disease	A:9.4 (3.6) C:8.3 (4.1)	−	Custom-made virtual reality balance training system	BBS; DGI; Timed Up-and-Go test; PDQ-39; UPDRS-III	Baseline Intervention 6 weeks Follow-up: 2 weeks	Post- treatment: 13.0% End of follow-up: 13.0%
14	Yen et al., 2011	China	T:42 A:14 (85.7%) C:14 (85.7%) Untrained control group: 14 (64.3%)	A:70.4 (6.5) C:70.1 (6.9) Untrained control group:71.6 (5.8)	UPDRS-III: A:15.1 (3.2) C:15.9 (2.4)	Parkinson’s Disease	A:6.0 (2.9) C:6.1 (3.3)	Dopamine agonists or dopamine replacement antiparkinsonian medications	Dimensional (3D) VR Balance Training System	SOT	Baseline Intervention 6 weeks Follow-up: 4 weeks	Post- treatment: 9.5% End of follow-up: 23.8%
15	Nanhoe-Mahabier et al., 2012	Netherlands	T:20 (80%) A:10 (80%) C: 10 (80%)	A:59.3 (2) C:58.6 (2.5)	UPDRS-III: A:17.9 (2.7) C:15.4 (1.1)	Parkinson’s Disease	A:3.7 (0.8) C:3.9 (0.8)	levodopa equivalent	Balance biofeedback system	Duration until completion of the walking tasks, the 90% range of pitch and roll sway angle and the 90% range of pitch and roll sway angular velocity	Baseline; post-training	None

SD, standard deviation; TUG, Timed Up and Go test, ABC, Activities-specific Balance Confidence scale; FOGQ, Freezing Of Gait Questionnaire; PDQ-39, Parkinson’s Disease Questionnaire-39; COP, Center of Pressure; BBT, Box Blocks Test; tfftte, Time from first touch to the end; atom, Average time of manipulation; IB, Inserted boxes; tnot, Total number of tries; ATI, Average tremor indicator; atips, Average tremor indicator per second; UPDRS, Unified Parkinson’s Disease Rating Scale; UPDRS-III, Unified Parkinson’s Disease Rating Scale—Motor Examination III; BBS, Berg Balance Scale; falls, number of falls in the previous month; 10MWT, 10-Meter Walking Test; DGI, Dynamic Gait Index; PDQ-8, Parkinson’s Disease Quality-of-Life questionnaire; UST, Unipedal Stance Test; FGA, functional gait assessment; DC, directional control; ME, maximal excursion; MV, movement velocity; SOT, sensory organization test; TE, traditional exercise; FES-I, Falls Efficacy Scale—International; TT, Tinetti Test; ADL, activities of daily living; IADL, instrumental activities of daily living; QOL-AD, quality of life-Alzheimer’s disease; MMSE, Mini-Mental State Examination; ST, Single task; DT, dual task; MFI, multidimensional fatigue inventory; HY, Hoehn and Yahr stage; PG, posture and gait subscore; FRT, functional reach test; HADS, hospital anxiety and depression scale; SOM, somatosensory; VIS, vision; VES, vestibular; PREF, preference. * The participants of the two articles were from the same population, but there were differences in the outcome measures between the two articles.

**TABLE 3:** Interventions in the included trials.

	Author, year	Intervention group FITT	Control group ITT	Adverse events
1	Carpinella et al., 2017	Frequency: 45-min sessions three times a week Intensity: mild Time: 20 sessions Type: balance and gait training with biofeedback Gamepad system (changing the reference values of the exercise, including more difficult tasks, changing the perceptive context) (e.g., altering proprioception through foam pads under feet), and/or including a dual task (e.g., walking holding a tray with a ball above)	Frequency: 45-min sessions three times a week Intensity: Mild Time: 20 sessions Type: Conventional physiotherapy [5 min of muscle stretching (hamstrings, quadriceps, and calves) and mobilization exercises (e.g., trunk rotation, hip abduction, flexion), followed by 40 min of balance and gait exercises similar to those performed by the experimental group]	Not mentioned
2	Cikajlo and Potisk, 2019	Frequency: 30-min sessions 10 times in 3 weeks Intensity: mild Time: 3 weeks Type: using immersive VR (3D) called “10Cubes” exergaming system to finish the VR task five times to pick and place the 10 virtual cubes in the virtual environment into the open treasure chest using the more affected hand.	Frequency: 30-min sessions 10 times in 3 weeks Intensity: Mild Time: 3 weeks Type: using a non-immersive environment (2D) (a laptop) to move to manipulate the virtual cubes.	None
3	Gandolfi et al., 2017	Frequency: 50 min 3 days/week Intensity: mild Time: 7 weeks + 1-month follow-up Type: in-home VR balance training called TeleWii consisted of 21 sessions of balance exercises to finish 10 exergames selected by the physiotherapist	Frequency: 50 min 3 days/week Intensity: Mild Time: 7 weeks + 1-month follow-up Type: in-clinic sensory integration balance training (SIBT) consisted of 21 sessions of balance and gait exercises under different sensory conditions (free vision, blindfolded, wearing a visual-conflict dome, firm/compliant surfaces, and neck extensions)	None
4	Kafle and Rizvi, 2021	Frequency: no information Intensity: no information Time: no information Type: using Nintendo Wii FitTM console.	Frequency: no information Intensity: no information Time: no information Type: global exercise	Not mentioned
5	Liao et al., 2015a	Frequency: 45-min sessions two times a week Intensity: moderate Time: 6 weeks + 1-month follow-up Type: virtual reality-based Wii Fit exercise included 10 min of yoga exercises, 15 min of strengthening exercises, and 20 min of balance games	Frequency: 45-min sessions two times a week Intensity: moderate Time: 6 weeks + 1-month follow-up Type: traditional exercise included 10 min of stretching exercises, 15 min of strengthening exercises, and 20 min of balance exercises	None
6	Liao et al., 2015b	Frequency: 45-min sessions two times a week Intensity: moderate Time: 6 weeks + 1-month follow-up Type: VR-based Wii Fit exercise included 10 min of yoga exercises, 15 min of strengthening exercises, and 20 min of balance games	Frequency: 45-min sessions two times a week Intensity: moderate Time: 6 weeks + 1-month follow-up Type: traditional exercise included 10 min of stretching exercises, 15 min of strengthening exercises, and 20 min of balance exercises	None
7	Padala et al., 2012	Frequency: 30-min sessions five times a week Intensity: moderate Time: 8 weeks Type: Wii Fit program included 10 min of yoga, 10 min of strength training, and 10 min of balance games.	Frequency: 30-min sessions five times a week Intensity: moderate Time: 8 weeks Type: walking group walked at their own pace as a group of three or four subjects at any given time with research personnel.	None
8	Pompeu et al., 2012	Frequency: 60-min sessions two times a week Intensity: moderate Time: 7 weeks + 60-day follow-up Type: 30 min on global exercises (10 min of warming, stretching, and active exercises; 10 min of resistance exercises for limbs; and 10 min of exercises in diagonal patterns for trunk, neck, and limbs) + 30 min on Wii-based motor and cognitive training (playing 10 Wii Fit games included static balance, dynamic balance, and stationary gait)	Frequency: 60-min sessions two times a week Intensity: moderate Time: 7 weeks + 60-day follow-up Type: 30 min on global exercises (10 min of warming, stretching, and active exercises; 10 min of resistance exercises for limbs; and 10 min of exercises in diagonal patterns for trunk, neck, and limbs) + 30 min on Wii-based motor and cognitive training (the same movements without the provision of external cues, feedback, and cognitive stimulation)	None
9	Ribas et al., 2017	Frequency: 30-min sessions two times a week Intensity: moderate Time:12 weeks + 60-day follow-up Type: seven Wii Fit games: Table Tilt, Tilt City, Penguin	Frequency: 30-min sessions two times a week Intensity: moderate Time: 12 weeks + 60-day follow-up Type: conventional exercise program: warming, stretching	
		Slide, Soccer Heading, Basic Run, Obstacle Course, and Basic Step (a Nintendo video game console with a Wii Balance Board)	and active exercises (10 min); resistance exercises for the limbs (10 min); and diagonal exercises for the trunk, neck, and limbs (10 min).	None
10	Santos et al., 2019	Frequency: 50 min a day, two times a week Intensity: moderate Time: 8 weeks Type: played four games in two sessions (Wii Sport and Wii Fit) standing up. In the first session, they played Boxing and Soccer Heading, and in the second session Golf and Running. Each session was performed for 20 min, with intervals of 1-min rest every 5 min of activity.	Frequency: 50 min a day, two times a week Intensity: moderate Time: 8 weeks Type: 30 min of specific diagonals [being superior limbs (flexion–abduction external rotation/extension–adduction–internal rotation); scapula (elevation and posterior depression), pelvis (anterior elevation/posterior depression), lower limbs (flexion–abduction–external rotation/internal extension–adduction–rotation) and 10 min of walking training in orthostasis or trunk extension training in ductus dorsal], with intervals of 1-min rest every 5 min of activity.	Not mentioned
11	Ugur and Sertel, 2020	Frequency: 30 min, two times a week Intensity: moderate Time: 6 weeks Type: training with games selected from different categories (soccer heading, tilt table, tightrope tension, perfect 10, cycling, tilt city, jogging plus, hula hoop, step basics, and penguin slide) with Nintendo Wii virtual reality device	Frequency: no clear Intensity: no clear Time: no clear Type: routine medical treatments	Not mentioned
12	van den Heuvel et al., 2014	Frequency: 60 min, two times a week Intensity: moderate Time: 5 weeks + 12-week follow-up Type: Visual feedback (VFT) training, the dynamic balance exercises focused on controlling body posture in the forward, backward, and sideward directions, exploring limits of stability, shifting weight from one foot to another, sit-to-stand movements, and included dual-task exercises.	Frequency: 60 min, two times a week Intensity: moderate Time: 5 weeks + 12-week follow-up Type: Conventional balance training, focused on training standing balance and included exercises while standing on one leg or with eyes closed, stepping exercises, dual-task exercises, sit-to-stand exercises, and exercises on the balancing beam or other challenging support surfaces.	None
13	Yang et al., 2016	Frequency: 50 min, two times a week Intensity: moderate Time: 6 weeks + 8-week follow-up Type: a 10-min warm-up stretching, three 10-min blocks of balance training [practiced the static posture maintaining with the VR balance training system (10 min) and dynamic weight shifting (2 × 10-min blocks)], and two 5-min breaks between blocks.	Frequency: 30 min, two times a week Intensity: moderate Time: 6 weeks + 8-week follow-up Type: In conventional balance training, participants practiced static posture maintaining (10-min block) and dynamic weight shifting (2 × 10-min blocks; Supplementary Appendix 2). The therapist in the control group guided the training and provided verbal instructions to correct the participants’ movements.	Not mentioned
14	Yen et al., 2011	Frequency: 30 min, two times a week Intensity: moderate Time: 6 weeks + 4-week follow-up Type: undergo 10 min of stretching exercises as a warm-up and 20-min VR challenges (including 10 min of the 3D ball-rolling game and 10 min of indoor-outdoor virtual activities.)	Frequency: 30 min, two times a week Intensity: moderate Time: 6 weeks + 4-week follow-up Type: Conventional balance training, undergo 10 min of stretching exercises and 20 min of intervention [(1) static stance, (2) dynamic weight shifting, and (3) external perturbations]	None
15	Nanhoe-Mahabier et al., 2012	12-task balance training with a real-time vibrotactile biofeedback, which was provided at a frequency of 250 Hz by eight vibrotactile sensors spaced equally around the headband. Activation thresholds were set at 40% of the 90% ranges of pitch and roll sway angular velocity derived during the second balance assessment of the first session, for each subject and for each task separately.	The same 12-task balance training without any biofeedback	Not mentioned

The included trials were carried out in China (*n* = 4), Brazil (*n* = 3), Italy (*n* = 2), Netherlands (*n* = 2), India (*n* = 1), Slovenia (*n* = 1), Turkey (*n* = 1), and the United States (*n* = 1) (Table 2). Nine studies used the Nintendo Wii Fit (Yen et al., 2011; Padala et al., 2012; Pompeu et al., 2012; Liao et al., 2015a,b; Gandolfi et al., 2017; Ribas et al., 2017; Ugur and Sertel, 2020; Kafle and Rizvi, 2021) connected to a large screen. Four studies (van den Heuvel et al., 2014; Yang et al., 2016; Cikajlo and Potisk, 2019; Santos et al., 2019) played sports games based on VR technology, one study (Carpinella et al., 2017) used a Gamepad system comprising six wearable inertial sensors, and one study (Nanhoe-Mahabier et al., 2012) conducted a balance training session with a real-time vibrotactile biofeedback system containing angular velocity sensors. One trial reported an immediate effect of one-time biofeedback program on trunk sway of PD (Nanhoe-Mahabier et al., 2012), while the rehabilitation process of other 14 trials was completed over 2 months with a minimum of 10 sessions and a maximum of 40 sessions. Besides, the dedicated time per training session ranged from 20 to 60 min; in three studies (Nanhoe-Mahabier et al., 2012; Santos et al., 2019; Kafle and Rizvi, 2021), the treatment time was not given. Regarding treatment frequency, most studies reported a frequency between two and five times a week, and only one study (Kafle and Rizvi, 2021) provided no information about intervention frequency. Two studies (Yang et al., 2016; Gandolfi et al., 2017) were carried out in the home circumstance, involving 99 participants.

### Risk of Bias and Quality of Evidence Appraisal

The risk of bias is reported in Supplementary Table 1. Most of the studies (more than 75%) present an unclear risk of bias in deviations from the intended interventions (effect of assignment to intervention). More than 50% included studies presented a low risk of bias in the randomization process, missing outcome data, measurement of the outcome, and selection of the reported result. The quality of evidence appraisal is given in Supplementary Table 2, and the quality ranged from low to moderate.

### Outcome Measurements

#### Primary Outcome: Balance

**FIGURE 2:**
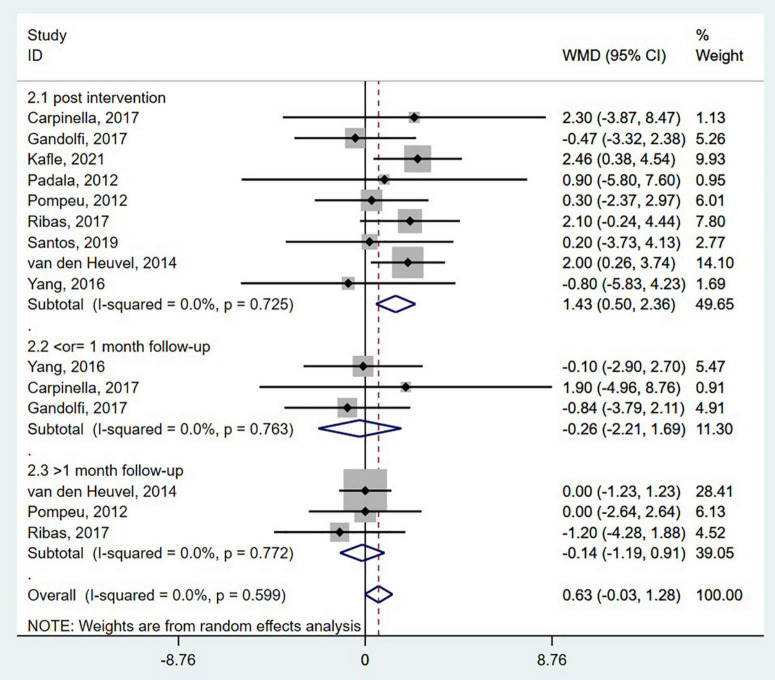
Forest plot of pooled results for the Berg balance scale.

The Berg balance scale is a comprehensive scale for postural stability assessment. Patients diagnosed with neurodegenerative diseases were assessed for static and dynamic postural control tasks using the BBS (Downs, 2015). The BBS was reported in nine studies (Padala et al., 2012; Pompeu et al., 2012; van den Heuvel et al., 2014; Yang et al., 2016; Carpinella et al., 2017; Gandolfi et al., 2017; Ribas et al., 2017; Santos et al., 2019; Kafle and Rizvi, 2021), with the duration from 3 to 12 weeks. When post-intervention data from nine randomized controlled studies were pooled, a significant improvement was found [WMD = 1.43; 95% CI, 0.50 to 2.36, *P* = 0.003; *I*^2^ = 0.0%, Figure 2(2.1)]. After 0.5–2 months of follow-up, no significant differences were found between the wearable sensor-based exercise and control [≤1-month follow-up, WMD = −0.26; 95% CI, −2.21 to 1.69, *P* = 0.79; >1-month follow-up, WMD = −0.14; 95% CI, −1.19 to 0.91, *P* = 0.80, Figures 2(2.2; 2.3)]. Besides, the overall result of this pooled analysis is not significant (WMD = 0.63; 95% CI, −0.03 to 1.28, *P* = 0.061; *I*^2^ = 0.0%, Figure 2). In addition, Egger’s test revealed no evidence of publication bias (*P* = 0.815).

**FIGURE 3:**
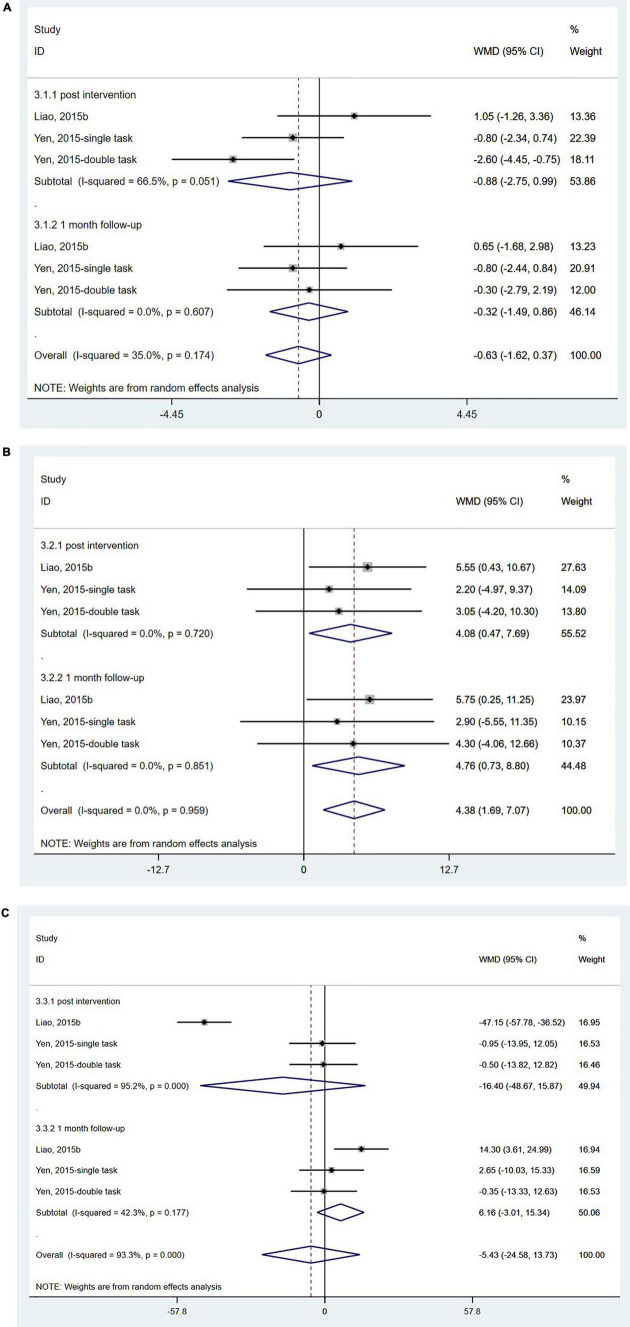
Forest plot of pooled results for sensory organization test. **(A)** Somatosensory scores. **(B)** Visual scores. **(C)** Vestibular scores.

The SOT is used to quantify sensory integration ability for balance using computerized dynamic posturography. It can provide information related to the integration of vision, somatosensory, and vestibular systems for maintaining postural stability (Gera et al., 2016). Only two studies (Yen et al., 2011; Liao et al., 2015b) from China with 78 participants used the SOT, and the results showed no significant effect of wearable sensor-based exercise on the somatosensory score (WMD = −0.63; 95% CI, −1.62 to 0.37, *P* = 0.216; *I*^2^ = 35.0%) (Figure 3A) or the vestibular score (WMD = −5.43; 95% CI, −24.58 to 13.73, *P* = 0.579; *I*^2^ = 93.3%) (Figure 3C). However, a significant change in visual score was found both post-assessment and at 1-month follow-up assessment (WMD = 4.38; 95% CI, 1.69 to 7.07, *P* = 0.001; *I*^2^ = 0.0%) (Figure 3B).

#### Secondary Outcomes

##### Motor Symptoms

**FIGURE 4:**
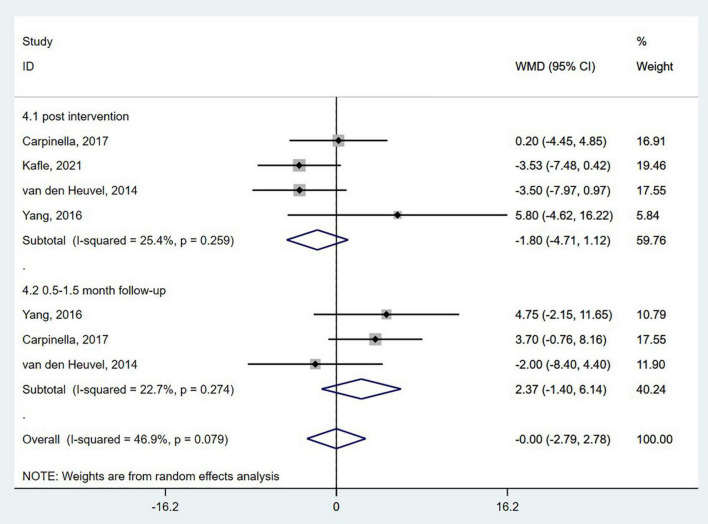
Forest plot of pooled results for Unified Parkinson’s Disease Rating Scale-III.

The UPDRS is the most used comprehensive scale of PD severity. The scale has four sub-scales: I = Motivation, Behavior, and Mood; II = ADL; III = Motor Examination; and IV = Complications of Therapy (Goetz et al., 2008). The pooled results of four studies (van den Heuvel et al., 2014; Yang et al., 2016; Carpinella et al., 2017; Kafle and Rizvi, 2021) did not show a significant effect of wearable sensor-based exercise on the UPDRS-III either at the post-intervention or 0.5–1.5-month follow-up (WMD = −0.00; 95% CI, −2.79 to 2.78, *P* = 0.997; *I*^2^ = 46.9%) (Figure 4).

##### Mobility Ability

**FIGURE 5:**
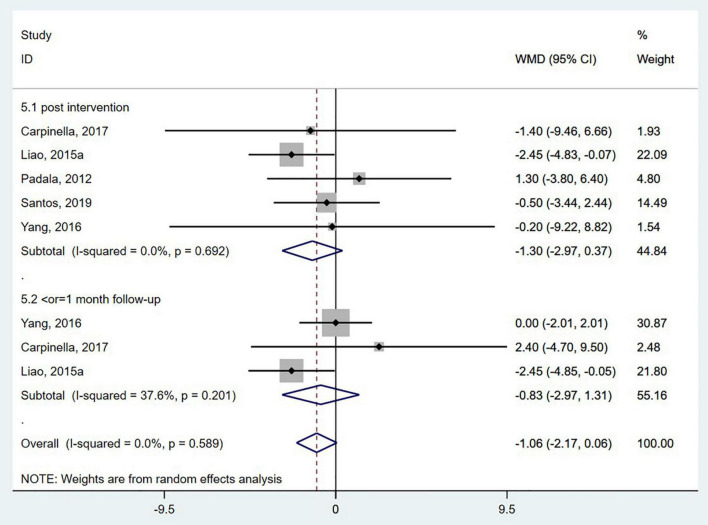
Forest plot of pooled results for timed “Up and Go” test.

**FIGURE 6:**
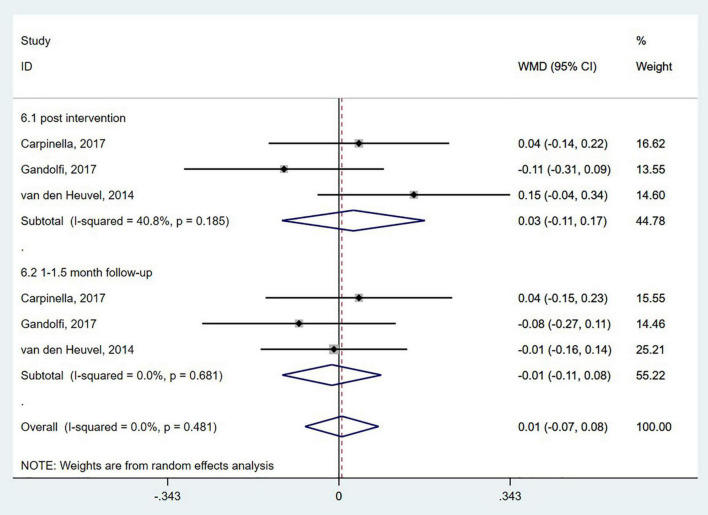
Forest plot of pooled results for 10-meter walking test.

Five studies (Padala et al., 2012; Liao et al., 2015a; Yang et al., 2016; Carpinella et al., 2017; Santos et al., 2019) used the TUG to evaluate functional mobility. In the TUG, the participants were timed as they stood up from a backrest chair, walked with a normal gait 3 meters forward, and then sat down and leaned back (de Oliveira Silva et al., 2019; Yoo et al., 2020). The pooled analysis showed no significant effect of wearable sensor-based exercise on the TUG test (WMD = −1.06; 95% CI, −2.17 to 0.06, *P* = 0.589; *I*^2^ = 0%) (Figure 5). Three studies (van den Heuvel et al., 2014; Carpinella et al., 2017; Gandolfi et al., 2017) measured the effects on functional mobility by the 10MWT. Our analysis revealed no significant effect of wearable sensor-based exercise on the 10MWT (WMD = 0.01; 95% CI, −0.07 to 0.08, *P* = 0.481; *I*^2^ = 0%) (Figure 6).

**FIGURE 7:**
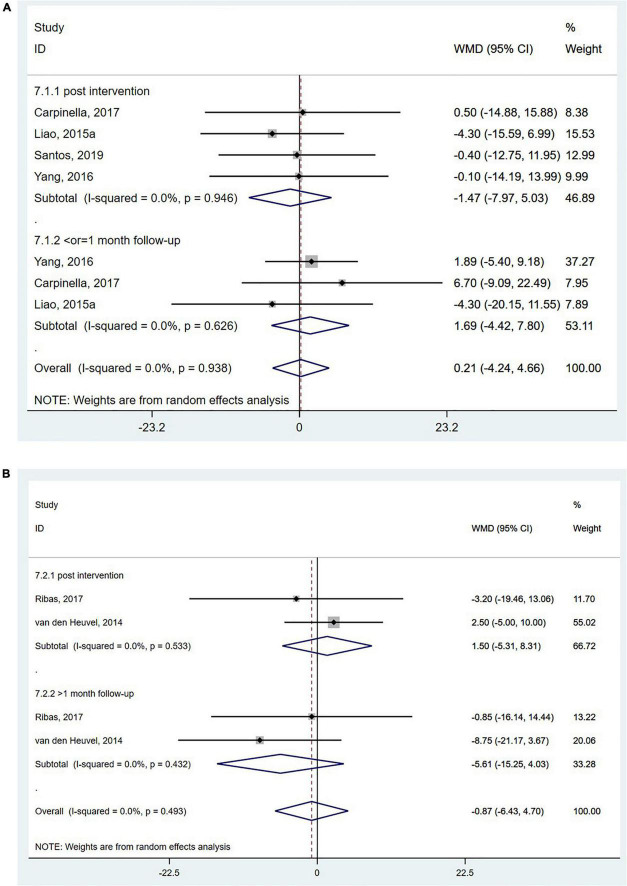
Forest plot of pooled results for **(A)** the total score of 39-item Parkinson’s disease questionnaire and **(B)** the mobility scores of the 39-item Parkinson’s disease questionnaire.

Parkinson’s disease questionnaire-39 is a self-reported quality-of-life questionnaire evaluating mobility and social functions in people with Parkinson’s disease based on a five-point Likert scale (Neff et al., 2018). The PDQ-39 was reported in four studies (Liao et al., 2015a; Yang et al., 2016; Carpinella et al., 2017; Santos et al., 2019), and the pooled analysis showed no significant effect of wearable sensor-based exercise on PDQ-39 scores (WMD = 0.21; 95% CI, −4.24 to 4.66, *P* = 0.926; *I^2^* = 0%, Figure 7A). The mobility scores of the PDQ-39 were reported in two studies (van den Heuvel et al., 2014; Ribas et al., 2017), and the pooled analysis did reveal no significant effect of wearable sensor-based exercise on these scores (WMD = −0.87; 95% CI, −6.43 to 4.70, *P* = 0.760; *I^2^* = 0%, Figure 7B; Peto et al., 2001).

##### Functional Gait Abilities

**FIGURE 8:**
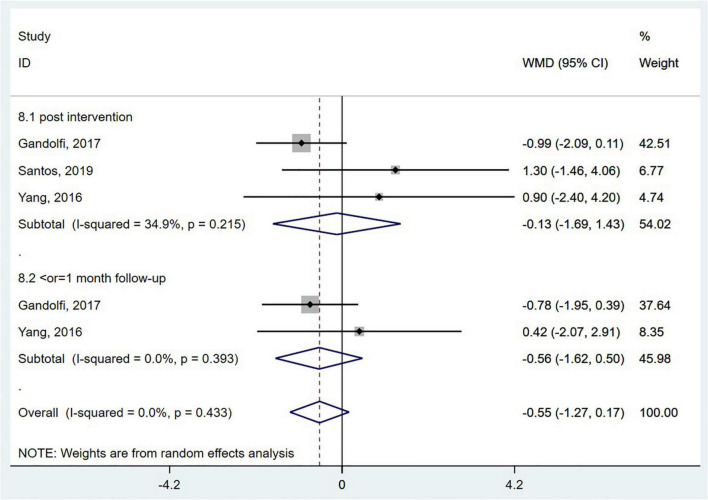
Forest plot of pooled results for dynamic gait index.

Dynamic gait index (DGI) has emerged as a valid indicator of functional gait abilities for people with balance and vestibular disorders. Scores on the DGI range from 0 to 24, with higher scores indicating better performance (Bloem et al., 2016). Three RCTs (Yang et al., 2016; Gandolfi et al., 2017; Santos et al., 2019) evaluated the effect of wearable sensor-based exercise on gait performance using the DGI. However, the pooled results did not reveal a significant effect (WMD = −0.55; 95% CI, −1.27 to 0.17, *P* = 0.135; *I^2^* = 0%) (Figure 8).

##### Fall-Associated Self-Efficacy

**FIGURE 9:**
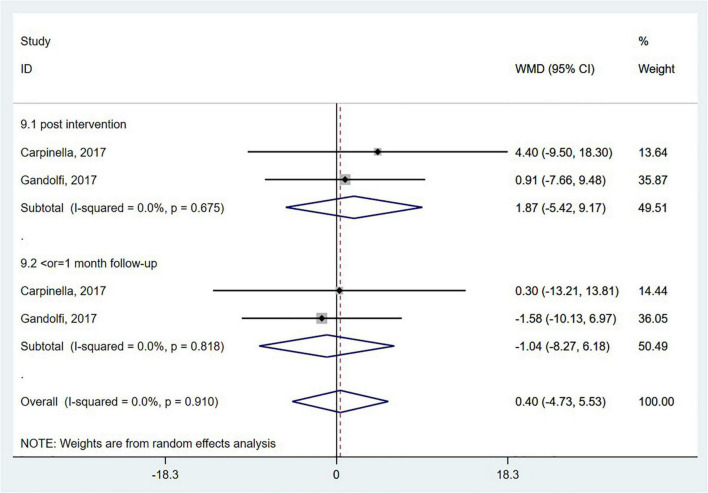
Forest plot of pooled results for activities-specific balance confidence scale.

Activities-specific balance confidence (ABC) scale measures balance confidence in particular postural control tasks. The ABC scale was employed in two studies (Carpinella et al., 2017; Gandolfi et al., 2017), and there was no significant difference between the wearable sensor-based exercise group and the control group (WMD = 0.40; 95% CI, −4.73 to 5.53, *P* = 0.879; *I^2^* = 0%, Figure 9).

##### Adverse Events and Acceptability

None of the included RCTs reported any adverse events, such as falling, during study duration. The dropout rate ranged from 0 to 23.5% (Tables 2, 3). One study evaluated the satisfaction of the subject with the wearable sensors, found most of the participants were positive about the device, and considered this a beneficial, reliable, easy to use, comfort, and safe technology (Carpinella et al., 2017).

## Discussion

This systematic review explored the effect of wearable sensor-based exercise on improving postural control in patients with neurodegenerative diseases compared with traditional exercise. The results of the meta-analysis including 14 studies indicated that the wearable sensor-based exercise could induce a significant short-term effect on balance, especially for an increased visual gain in the SOT. However, the 3–12-week wearable sensor-based exercise did not seem to improve mobility ability, functional gait abilities, and fall-associated self-efficacy, as the changes did not reach statistical significance. Overall, the wearable sensor-based exercise increases balance in patients with neurodegenerative diseases, but the evidence is lacking to improve postural control during daily activities, such as walking.

The movement disorders associated with neurodegeneration may be explained by various perspectives, such as oxidative stress or mitochondrial dysfunction (Borsche et al., 2021; Dionísio et al., 2021). Due to the complexity of neurodegeneration with many potential intervention targets, the research progress of drug treatment is slow, and the current drug treatment mainly focuses on improving motor symptoms (Armstrong and Okun, 2020; Dar et al., 2020). In contrast, an exercise intervention is a more direct protocol to improve motor symptoms, and the benefits of intervention can also delay the speed of neurodegeneration and improve the quality of life (Bonavita, 2020; Sujkowski et al., 2022). However, traditional exercise interventions often require the involvement of specialists, such as rehabilitation physicians and physical therapists. The quality of the intervention often depends on the experience of the specialists and the patient’s compliance (Botros et al., 2019; Schootemeijer et al., 2020). The involvement of sensor-based equipment brings new possibilities for an exercise intervention (Jahn et al., 2019). The instant biofeedback provided by sensors and the combination with exergame can form a standardized and interesting exercise protocol for patients with neurodegenerative movement disorders (Ribas et al., 2017; Ugur and Sertel, 2020; Kafle and Rizvi, 2021), such as avoiding obstacles or picking fruit.

The wearable sensors are directly attached to the patients to provide immediate and precise biofeedback on the patient’s movements (Albán-Cadena et al., 2021). Optical sensors often require a bright environment, and reflective clothing will affect the accuracy of their motion capture (Warmerdam et al., 2020). Optical sensors also need a wide living room or hospital treatment room to maximize the exercise experience, which may prevent some patients from using optical sensors. Compared with the use of optical sensors, such as cameras, the application environment of wearable sensors is rarely limited. In addition, patients can be trained in a home environment under the remote guidance of professionals with the emergence of commercialized sensor devices (Garcia-Agundez et al., 2019; Milosevic et al., 2020). It can be used as a feasible solution for an exercise intervention under epidemic conditions.

Although the neurodegenerative disease is progressive and the gray matter volume changes in the left inferior parietal cortex, middle temporal gyrus and right anterior precuneus were associated with the balance capacities (Sehm et al., 2014), a significant improvement in balance was found in the wearable sensor-based exercise group at the end of the intervention, which may be due to the real-time feedback and wrong action corrections in the intensive body control training in the protocol. This systematic review showed that most movement modalities in the interventional group used a center of gravity shifting training method, requiring patients to complete the directional and quantitative shifting of the center of gravity (Liao et al., 2015a,b; Yang et al., 2016; Santos et al., 2019). The inertial sensor placed on the waist can directly reflect the change in the center of gravity, while the pressure sensor placed on the sole can indirectly reflect the change in the center of gravity through the offset of the plantar pressure, thereby providing accurate and immediate biofeedback for the patient (Padala et al., 2012; Pompeu et al., 2012; van den Heuvel et al., 2014; Liao et al., 2015a,b; Carpinella et al., 2017; Ribas et al., 2017).

Otherwise, two studies in this systematic review used the SOT and demonstrated a significantly increased score in visual domain, which might owe to the contribution of visual input significantly increased during the wearable sensor-based exercise, compared with the traditional exercises (Yen et al., 2011; Liao et al., 2015b). The increased visual gain in the SOT conditions also reflects the importance of visual input for the control of balance. As balance perturbations tend to occur in dynamic tasks and in response to environmental constraints not present during the SOT, the SOT may provide additional information for clinical evaluation on neurodegenerative disease and deficient sensory processing (Chien et al., 2014).

Previous meta-analysis and systematic reviews concluded promising short- and long-term benefits of exercise on various meaningful outcomes, such as balance, gait, muscle strength, motor, and functional performance (Tomlinson et al., 2012; Shen et al., 2016; Mak et al., 2017). However, compared with the traditional exercise programs, the results for the secondary outcomes showed no significance in the mobility ability, functional gait abilities, and fall-associated self-efficacy in the wearable sensor-based exercise group. Impaired flexibility and muscle weakness are two common problems that may affect postural control and mobility. As compared to neurologically normal adults, people with neurodegenerative disease had a reduced range of trunk motion, which could partially be explained by axial muscle rigidity (Schenkman et al., 2001), with a reduction in muscle strength by 30–50% (Inkster et al., 2003).

Furthermore, limited by the technical conditions of the sensor as a piece of external equipment, the training protocol in the included studies mainly consisted of the control tasks with a small range of body sway. There was a lack of activities of daily living training that simulate daily scenes, such as walking (Carpinella et al., 2017; Cikajlo and Potisk, 2019). In this meta-analysis, the intervention duration was 3–12 weeks, and the negative results may be associated with overall short treatment durations with a relatively low frequency (an average of 6–7 weeks with two–three times a week). These treatment durations would be considered short relative to multimodal physical therapy for Parkinson’s disease (Tomlinson et al., 2012). On the other hand, although these measurements are the most frequently assessed in clinical practices, they might not have been sensitive enough to detect specific wearable sensor-based exercise training-related changes in dynamic postural control. Moreover, we did not find evidence that factors, such as control group (i.e., usual care vs. conventional training), sensor type, and type of training paradigm, were related to these negative findings, and the limited number of RCTs did not allow to draw definitive conclusions.

People with neurodegenerative diseases are nearly two times as likely to experience a fall as a healthy older person, often leading to debilitating effects on confidence, activity levels, and quality of life (Ashburn et al., 2019). A meta-analysis showed that performance confidence in overcoming barriers to exercise was best addressed with longer-term strategies that provided time for people to experience successfully conquering such barriers over a longer period (Higgins et al., 2014). With regard to the fall-associated self-efficacy in patients with neurodegenerative diseases, the results of our systematic review also showed that the 3–12-week wearable sensor-based exercise cannot improve fall-associated self-efficacy significantly, which might be due to the relatively short intervention duration. Moreover, people with neurodegenerative diseases may suffer from anxiety and depression, which could negatively impact self-efficacy (Stevens et al., 2020), and also, the community and societal factors may account for an important proportion of the improvement in self-efficacy (Bellou et al., 2016; Lee et al., 2016; Rosa Silva et al., 2020). Multidisciplinary teams, such as exercise specialists, occupational therapists, and psychologists, should take cognitive and psychological symptoms into account when working with these patients to maximize the potential effectiveness of treatment, and social workers may be needed to help patients with neurodegenerative diseases reintegrate into the community and society (Stożek et al., 2016; Homayoun, 2018; Ritter and Bonsaksen, 2019). Future research is recommended to construct a comprehensive intervention system for fall-associated self-efficacy.

### Strengths and Limitations

The strength of this meta-analysis is that only RCTs were included. Furthermore, to the best of our knowledge, this is the first meta-analysis to focus on postural control ability in patients with neurodegenerative diseases compared with traditional therapeutic exercise. However, several limitations need to be highlighted in this systematic review. First, as an indicator of postural stability, the BBS has been significantly improved, but whether the degree of postural stability improvement can lead to effective clinical improvement in movement disorders is unclear. Second, due to the insufficient number of included studies, no significant improvement was found in indicators related to social life functions, such as activities of daily living and quality of life. Finally, the quality of the evidence in this article ranged from moderate to very low (Supplementary Table S2). Therefore, caution should be applied, however, to avoid overestimation of findings given the several methodological weaknesses in available studies, such as short follow-up (<12 months), small sample size (*n* < 100), and missed evaluation of facilitator.

## Conclusion

Compared with traditional exercise interventions, the wearable sensor-based exercise can significantly improve balance in patients with neurodegenerative diseases. However, there was still a lack of evidence showing the superiority of wearable sensor-based exercise technology for other indicators of dynamic postural control, such as motor symptoms, mobility ability, functional gait abilities, and fall-associated self-efficacy. In future, it is necessary to conduct more research on the effect of wearable sensor-based exercise on sensory organization and try to build a comprehensive rehabilitation treatment system to improve both postural control and quality of life.

## Data Availability Statement

All raw data included in this study are available upon request by contact with the corresponding author.

## Author Contributions

XL and QD had the original idea. XL, ZC, and MZ performed the literature search. QD and HG screened the studies using the eligibility criteria and undertook the data collection. XZ and JT assessed the risk of bias. XL, YY, and SG analyzed, interpreted, and discussed the results. All authors contributed to the article and approved the submitted version.

## Conflict of Interest

The authors declare that the research was conducted in the absence of any commercial or financial relationships that could be construed as a potential conflict of interest.

## Publisher’s Note

All claims expressed in this article are solely those of the authors and do not necessarily represent those of their affiliated organizations, or those of the publisher, the editors and the reviewers. Any product that may be evaluated in this article, or claim that may be made by its manufacturer, is not guaranteed or endorsed by the publisher.
