# Supplementary material for: Effect of Wearable Sensor-Based Exercise on Musculoskeletal Disorders in Individuals With Neurodegenerative Diseases: A Systematic Review and Meta-Analysis
Source: Front Aging Neurosci. 2022 Jul 26;14:934844. doi: 10.3389/fnagi.2022.934844 (PMC9360755; doi:10.3389/fnagi.2022.934844)
Supplement: Supplementary file 1 [file Data_Sheet_1.docx]

**Effect of Wearable Sensor-Based Exercise on Postural Control Ability in Neurodegenerative Musculoskeletal Patients:**

**A Systematic Review and Meta-Analysis**

**1. PubMed**

#1 “Single-Blind Method”[Mesh] OR “Double-Blind Method”[Mesh] OR “Randomized Controlled Trials as Topic”[Mesh] OR “Randomized Controlled Trial”[Publication Type] OR “Intention to Treat Analysis”[Mesh] OR “Controlled Clinical Trials as Topic”[Mesh] OR “Clinical Trials as Topic”[Mesh] OR “Clinical Trial”[Publication Type] OR randomized controlled trial[Publication Type]

#2 “random*”[Title/Abstract] OR allocation[Title/Abstract] OR “random allocation”[Title/Abstract] OR placebo[Title/Abstract] OR single blind[Title/Abstract] OR double blind[Title/Abstract] OR “randomized controlled trial*”[Title/Abstract] OR RCT[Title/Abstract]

#3 #1 OR #2

#4 animals NOT humans

#5 #3 NOT #4

#6 “Parkinson Disease”[Mesh] OR “Parkinson*”[Title/Abstract] OR “PD”[Title/Abstract] OR “Paralysis Agitans”[Title/Abstract] OR “Alzheimer Disease”[Mesh] OR “Alzheimer”[Title/Abstract] OR “ATD”[Title/Abstract] OR “Dementia, Senile”[Title/Abstract] OR “Senile Dementia”[Title/Abstract] OR “Primary Senile Degenerative Dementia”[Title/Abstract] OR “Dementia, Primary Senile Degenerative”[Title/Abstract] OR “Dementia, Presenile”[Title/Abstract] OR “Presenile Dementia”[Title/Abstract] OR “Amyotrophic Lateral Sclerosis”[Mesh] OR “Sclerosis, Amyotrophic Lateral”[Title/Abstract] OR “ALS”[Title/Abstract] OR “motor neuron diseases”[Title/Abstract] OR “Gehrig's Disease”[Title/Abstract] OR “Gehrig Disease”[Title/Abstract] OR “Charcot Disease”[Title/Abstract] OR “Guam Disease”[Title/Abstract] OR “Disease, Guam”[Title/Abstract]

#7 “Biofeedback”[Title/Abstract] OR “Sensor*”[Title/Abstract] OR “Inertial”[Title/Abstract] OR “IMU”[Title/Abstract] OR “Accelerometry”[Mesh] OR “Acceleromet*”[Title/Abstract] OR “Actigraph*”[Title/Abstract] OR “Gyroscope*”[Title/Abstract] OR “Magnetometer*”[Title/Abstract] OR “Virtual Reality”[Mesh] OR “Virtual Reality”[Title/Abstract] OR “Motion Sensing Game*”[Title/Abstract] OR “Active-Play Video Game*”[Title/Abstract] OR “Xbox”[Title/Abstract] OR “Wii”[Title/Abstract] OR “Kinect”[Title/Abstract] OR “Exergaming”[Mesh] OR “Exergam*”[Title/Abstract]

#8 “Rehabilitation”[Mesh] OR “Rehabilitation*”[Title/Abstract] OR “Physiotherapy”[Title/Abstract] OR “Physical Therapy”[Title/Abstract] OR “Exercise”[Mesh] OR “Exercise*”[Title/Abstract] OR “Physical Activity”[Title/Abstract] OR “Sports”[Mesh] OR “Sport*”[Title/Abstract] OR “Training”[Title/Abstract]

#9 “Postural Balance”[Mesh] OR “Balance”[Title/Abstract] OR “Equilibrium”[Title/Abstract] OR “Stability”[Title/Abstract] OR “Posture”[Mesh] OR “Postur*”[Title/Abstract] OR “Motor Control”[Title/Abstract] OR “Proprioception”[Mesh] OR “Propriocepti*”[Title/Abstract] OR “Somatosensory”[Title/Abstract] OR “Sensorimotor”[Title/Abstract] OR “Neuromuscular”[Title/Abstract] OR “Neuromotor”[Title/Abstract] OR “Coordination”[Title/Abstract] OR “Muscle Activation”[Title/Abstract] OR “Electromyography”[Mesh] OR “Electromyography”[Title/Abstract] OR “EMG”[Title/Abstract] OR “Kinematics”[Title/Abstract] OR “Force Plate”[Title/Abstract] OR “Center of Pressure”[Title/Abstract] OR “COP”[Title/Abstract] OR “Muscle Activity”[Title/Abstract] OR “Gait”[Mesh] OR “Gait”[Title/Abstract] OR “Walking”[Mesh] OR “Walk*”[Title/Abstract]

#10 #5 AND #6 AND #7 AND #8 AND #9

**2. EMBASE:**

#1 ‘randomization’/exp OR ‘placebo’/exp OR ‘placebo effect’/exp OR ‘single blind procedure’/exp OR ‘double blind procedure’/exp OR ‘randomized controlled trial’/exp OR ‘randomized controlled trial (topic)’/exp OR ‘controlled clinical trial’/exp OR ‘controlled clinical trial (topic)’/exp OR ‘clinical trial’/exp OR ‘clinical trial (topic)’/exp

#2 random*:ab,ti OR allocation:ab,ti OR ‘random allocation’:ab,ti OR placebo:ab,ti OR ‘single blind’:ab,ti OR ‘double blind’:ab,ti OR ‘randomised controlled trial*’:ab,ti OR ‘randomized controlled trial*’:ab,ti OR RCT:ab,ti OR ‘clinical trial*’:ab,ti

#3 #1 OR #2

#4 ‘Parkinson Disease’/exp OR ‘Parkinson*’:ab,ti OR ‘PD’:ab,ti OR ‘Paralysis Agitans’:ab,ti OR ‘Alzheimer Disease’/exp OR ‘Alzheimer’:ab,ti OR ‘motor neuron diseases’/exp OR ‘Dementia, Senile’:ab,ti OR ‘Senile Dementia’:ab,ti OR ‘Primary Senile Degenerative Dementia’:ab,ti OR ‘Dementia, Primary Senile Degenerative’:ab,ti OR ‘Dementia, Presenile’:ab,ti OR ‘Presenile Dementia’:ab,ti OR ‘Diffuse Cortical Sclerosis’:ab,ti OR ‘Amyotrophic Lateral Sclerosis’/exp OR ‘Sclerosis, Amyotrophic Lateral’:ab,ti OR ‘ALS’:ab,ti OR ‘Gehrig Disease’:ab,ti OR ‘Gehrigs Disease’:ab,ti OR ‘Charcot Disease’:ab,ti OR ‘Guam Disease’:ab,ti OR ‘Disease, Guam’:ab,ti OR ‘Lou-Gehrigs Disease’:ab,ti OR ‘Disease, Lou-Gehrigs’:ab,ti

#5 ‘Biofeedback’/exp OR ‘Biofeedback’:ab,ti OR ‘Sensor’/exp OR ‘Sensor*’:ab,ti OR ‘Inertial’:ab,ti OR ‘IMU’:ab,ti OR ‘Accelerometry’/exp OR ‘Acceleromet*’:ab,ti OR ‘Actigraph*’:ab,ti OR ‘Gyroscope*’:ab,ti OR ‘Magnetometer*’:ab,ti OR ‘Virtual Reality’/exp OR ‘Virtual Reality’:ab,ti OR ‘Motion Sensing Game*’:ab,ti OR ‘Active-Play Videogame*’:ab,ti OR ‘Active-Play Video Game*’:ab,ti OR ‘Xbox’:ab,ti OR ‘Wii’:ab,ti OR ‘Kinect’:ab,ti OR ‘Exergam*’:ab,ti

#6 ‘Rehabilitation’/exp OR ‘Rehabilitation*’:ab,ti OR ‘Physiotherapy’/exp OR ‘Physiotherapy’:ab,ti OR ‘Physical Therapy’:ab,ti OR ‘Exercise’/exp OR ‘Exercise*’:ab,ti OR ‘Physical Activity’/exp OR ‘Physical Activity’:ab,ti OR ‘Sport’/exp OR ‘Sport*’:ab,ti OR ‘Training’:ab,ti

#7 ‘Body Equilibrium’/exp OR ‘Balance’:ab,ti OR ‘Equilibrium’:ab,ti OR ‘Stability’:ab,ti OR ‘Body Position’/exp OR ‘Postur*’:ab,ti OR ‘Motor Control’/exp OR ‘Motor Control’:ab,ti OR ‘Proprioception’/exp OR ‘Propriocepti*’:ab,ti OR ‘Somatosensory’:ab,ti OR ‘Sensorimotor Function’/exp OR ‘Sensorimotor’:ab,ti OR ‘Neuromuscular’:ab,ti OR ‘Neuromotor’:ab,ti OR ‘Coordination’:ab,ti OR ‘Muscle Activation’:ab,ti OR ‘Electromyography’/exp OR ‘Electromyography’:ab,ti OR ‘EMG’:ab,ti OR ‘Kinematics’/exp OR ‘Kinematics’:ab,ti OR ‘Force Plate’:ab,ti OR ‘Center of Pressure’:ab,ti OR ‘COP’:ab,ti OR ‘Muscle Activity’:ab,ti OR ‘Walking’/exp OR ‘Gait’:ab,ti OR ‘Walk*’:ab,ti

#8 #3 AND #4 AND #5 AND #6 AND #7

**3. Cochrane Library**

#1 random*:ti,ab,kw OR allocation:ti,ab,kw OR placebo:ti,ab,kw OR (“single blind”):ti,ab,kw OR (“double blind”):ti,ab,kw OR ("randomized controlled trial*"):ti,ab,kw OR RCT:ti,ab,kw OR ("clinical trial*"):ti,ab,kw

#2 (randomized controlled trial):pt OR (clinical trial):pt

#3 #1 OR #2

#4 MeSH descriptor: [Parkinson Disease] explode all trees

#5 MeSH descriptor: [Alzheimer Disease] explode all trees

#6 MeSH descriptor: [Amyotrophic Lateral Sclerosis] explode all trees

#7 “Parkinson*”:ti,ab,kw OR “PD”:ti,ab,kw OR “Paralysis Agitans”:ti,ab,kw OR “Alzheimer”:ti,ab,kw OR “ATD”:ti,ab,kw OR “Dementia, Senile”:ti,ab,kw OR “Senile Dementia”:ti,ab,kw OR “Primary Senile Degenerative Dementia”:ti,ab,kw OR “Dementia, Primary Senile Degenerative”:ti,ab,kw OR “Dementia, Presenile”:ti,ab,kw OR “Presenile Dementia”:ti,ab,kw OR “Sclerosis, Amyotrophic Lateral”:ti,ab,kw OR “ALS”:ti,ab,kw OR “Gehrig’s Disease”:ti,ab,kw OR “Gehrig Disease”:ti,ab,kw OR “Gehrigs Disease”:ti,ab,kw OR “Charcot Disease”:ti,ab,kw OR “Guam Disease”:ti,ab,kw OR “Disease, Guam”:ti,ab,kw OR “motor neuron diseases”:ti,ab,kw

#8 #4 OR #5 OR #6 OR #7

#9 MeSH descriptor: [Accelerometry] explode all trees

#10 MeSH descriptor: [Virtual Reality] explode all trees

#11 MeSH descriptor: [Exergaming] explode all trees

#12 “Biofeedback”:ti,ab,kw OR “Sensor*”:ti,ab,kw OR “Inertial”:ti,ab,kw OR “IMU”:ti,ab,kw OR “Acceleromet*”:ti,ab,kw OR “Actigraph*”:ti,ab,kw OR “Gyroscope*”:ti,ab,kw OR “Magnetometer*”:ti,ab,kw OR “Virtual Reality”:ti,ab,kw OR “Motion Sensing Game*”:ti,ab,kw OR “Xbox”:ti,ab,kw OR “Wii”:ti,ab,kw OR “Kinect”:ti,ab,kw OR “Exergam*”:ti,ab,kw

#13 #9 OR #10 OR #11 OR #12

#14 MeSH descriptor: [Rehabilitation] explode all trees

#15 MeSH descriptor: [Exercise] explode all trees

#16 MeSH descriptor: [Sports] explode all trees

#17 “Rehabilitation*”:ti,ab,kw OR “Physiotherapy”:ti,ab,kw” OR “Physical Therapy”:ti,ab,kw” OR “Exercise*”:ti,ab,kw OR “Physical Activity”:ti,ab,kw OR “Sport*”:ti,ab,kw OR “Training”:ti,ab,kw

#18 #14 OR #15 OR #16 OR #17

#19 MeSH descriptor: [Postural Balance] explode all trees

#20 MeSH descriptor: [Posture] explode all trees

#21 MeSH descriptor: [Proprioception] explode all trees

#22 MeSH descriptor: [Electromyography] explode all trees

#23 MeSH descriptor: [Gait] explode all trees

#24 MeSH descriptor: [Walking] explode all trees

#25 “Balance”:ti,ab,kw OR “Equilibrium”:ti,ab,kw OR “Stability”:ti,ab,kw OR “Postur*”:ti,ab,kw OR “Motor Control”:ti,ab,kw OR “Propriocepti*”:ti,ab,kw OR “Somatosensory”:ti,ab,kw OR “Sensorimotor”:ti,ab,kw OR “Neuromuscular”:ti,ab,kw OR “Neuromotor”:ti,ab,kw OR “Coordination”:ti,ab,kw OR “Muscle Activation”:ti,ab,kw OR “Electromyography”:ti,ab,kw OR “EMG”:ti,ab,kw OR “Kinematics”:ti,ab,kw OR “Force Plate”:ti,ab,kw OR “Center of Pressure”:ti,ab,kw OR “COP”:ti,ab,kw OR “Muscle Activity”:ti,ab,kw OR “Gait”:ti,ab,kw OR “Walk*”:ti,ab,kw

#26 #19 OR #20 OR #21 OR #22 OR #23 OR #24 OR #25

#27 #3 AND #8 AND #13 AND #18 AND #26

**4. Web of Science**

#1 TS=(“random*” OR “allocation” OR “random allocation” OR “placebo” OR “single blind” OR “single blind method” OR “double blind” OR “double blind method” OR “randomized controlled trial*” OR “randomised controlled trial*” OR “RCT” OR “clinical trial*”)

#2 TS=(“Parkinson*” OR “PD” OR “Paralysis Agitans” OR “Alzheimer” OR “ATD” OR “Dementia, Senile” OR “Senile Dementia” OR “Primary Senile Degenerative Dementia” OR “Dementia, Primary Senile Degenerative” OR “Dementia, Presenile” OR “Presenile Dementia” OR “Sclerosis, Amyotrophic Lateral” OR “ALS” OR “Gehrig’s Disease” OR “Gehrig Disease” OR “Gehrigs Disease” OR “Charcot Disease” OR “Guam Disease” OR “Disease, Guam” OR “motor neuron diseases” OR “Lou-Gehrigs Disease” OR “Disease, Lou-Gehrigs”)

#3 TS=(“Biofeedback” OR “Sensor*” OR “Inertial” OR “IMU” OR “Acceleromet*” OR “Actigraph*” OR “Gyroscope*” OR “Magnetometer*” OR “Virtual Reality” OR “Motion Sensing Game*” OR “Active-Play Videogame*” OR “Active-Play Video Game*” OR “Xbox” OR “Wii” OR “Kinect” OR “Exergam*”)

#4 TS=(“Rehabilitation*” OR “Physiotherapy” OR “Exercise*” OR “Physical Activity” OR “Sport*” OR “Training”)

#5 TS=(“Balance” OR “Equilibrium” OR “Stability” OR “Postur*” OR “Motor Control” OR “Propriocepti*” OR “Somatosensory” OR “Sensorimotor” OR “Neuromuscular” OR “Neuromotor” OR “Coordination” OR “Muscle Activation” OR “Electromyography” OR “EMG” OR “Kinematics” OR “Force Plate” OR “Center of Pressure” OR “COP” OR “Muscle Activity” OR “Gait” OR “Walk*”)

#6 #1 AND #2 AND #3 AND #4 AND #5

Timespan=All years. Databases=core collection

**5. CINAHL (Ebsco)**

S1 MH(“Random Assignment” OR “Placebo*” OR “Placebo Effect” OR “Single-Blind Studies” OR “Double-Blind Studies” OR “Triple-Blind Studies” OR “Randomized Controlled Trials” OR “comparative studies” OR “Evaluation Research” OR “Prospective Studies” OR “crossover Design” OR “Prospective Studies” OR “Clinical Trials” OR “Clinical Trial Registry”)

S2 TX(“random$” OR “allocation” OR “random allocation” OR “placebo$” OR “single blind” OR “double blind” OR “randomi?ed controlled trial*” OR “controlled clinical trial*” OR “comparative study” OR “evaluation stud*” OR “follow-up stud*” OR “cross-over stud*” OR “control$” OR “RCT” OR “clinical trial*”)

S3 PT( “randomized controlled trial” OR “clinical trial*”)

S4 S1 OR S2 OR S3

S5 MH Animals NOT MH Human

S6 S4 NOT S5

S7 MH(“Parkinson Disease” OR “Alzheimer's Disease” OR “Amyotrophic Lateral Sclerosis”) OR AB(“Parkinson*” OR “PD” OR “Paralysis Agitans” OR “Alzheimer” OR “ATD” OR “Dementia, Senile” OR “Senile Dementia” OR “Primary Senile Degenerative Dementia” OR “Dementia, Primary Senile Degenerative” OR “Dementia, Presenile” OR “Presenile Dementia” OR “Sclerosis, Amyotrophic Lateral” OR “ALS” OR “Gehrig’s Disease” OR “Gehrig Disease” OR “Gehrigs Disease” OR “Charcot Disease” OR “motor neuron diseases” OR “Guam Disease” OR “Disease, Guam” OR “Lou-Gehrigs Disease” OR “Disease, Lou-Gehrigs”)

S8 MH(“Rehabilitation+” OR “Exercise+” OR “Physical Activity” OR “Sports+”) OR AB(“Rehabilitation*” OR “Physiotherapy” OR “Physical Therapy” OR “Exercise*” OR “Physical Activity” OR “Sport*” OR “Training”)

S9 MH(“Posture+” OR “Proprioception+” OR “Electromyography” OR “Kinematics” OR “Walking+”) OR AB(“Balance” OR “Equilibrium” OR “Stability” OR “Postur*” OR “Motor Control” OR “Propriocepti*” OR “Somatosensory” OR “Sensorimotor” OR “Neuromuscular” OR “Neuromotor” OR “Coordination” OR “Muscle Activation” OR “Electromyography” OR “EMG” OR “Kinematics” OR “Force Plate” OR “Center of Pressure” OR “COP” OR “Muscle Activity” OR “Gait” OR “Walk*”)

S10 S6 AND S7 AND S8 AND S9

Limiters - Abstract Available; Peer Reviewed; Exclude MEDLINE records; Human; Language: English; Age Groups: All Adult
