# Supplementary material for: Effect of Wearable Sensor-Based Exercise on Musculoskeletal Disorders in Individuals With Neurodegenerative Diseases: A Systematic Review and Meta-Analysis
Source: Front Aging Neurosci. 2022 Jul 26;14:934844. doi: 10.3389/fnagi.2022.934844 (PMC9360755; doi:10.3389/fnagi.2022.934844)
Supplement: Supplementary file 2 [file Table_1.DOCX]

| **Effect of Wearable Sensor-based Exercise on Postural Control Ability in Neurodegenerative Musculoskeletal Patients: A Systematic Review and Meta-Analysis** | | | | | | |
| --- | --- | --- | --- | --- | --- | --- |
| **Patient or population:** patients with Neurodegenerative Musculoskeletal **Intervention:** Effect of Wearable Sensor-based Exercise on Postural Control Ability | | | | | | |
| **Outcomes** | **Illustrative comparative risks* (95% CI)** | | **Relative effect (95% CI)** | **No of Participants (studies)** | **Quality of the evidence (GRADE)** | **Comments** |
|  | Assumed risk | Corresponding risk |  |  |  |  |
|  | **Control** | **Effect of Wearable Sensor-based Exercise on Postural Control Ability** |  |  |  |  |
| **Berg Balance Scale (BBS)** Questionnaire |  | The mean berg balance scale (BBS) in the intervention groups was **1.431 higher** (0.502 to 2.359 higher) |  | 328 (9 studies) | ⊕⊕⊕⊝ **moderate**^1^ |  |
| **Dynamic Gait Index (DGI)** Questionnaire |  | The mean dynamic gait index (DGI) in the intervention groups was **0.538 lower** (1.516 lower to 0.44 higher) |  | 126 (3 studies) | ⊕⊕⊕⊝ **moderate**^1^ |  |
| **Unified Parkinson Disease Rating Scale - Motor Examination III (UPDRS-3)** Questionnaire |  | The mean unified parkinson disease rating scale - motor examination Ⅲ (UPDRS-Ⅲ) in the intervention groups was **1.998 lower** (4.425 lower to 0.428 higher) |  | 150 (4 studies) | ⊕⊕⊕⊝ **moderate**^1^ |  |
| **Time Up and Go test (TUG)** |  | The mean time up and go test (TUG) in the intervention groups was **1.005 lower** (2.645 lower to 0.636 higher) |  | 133 (5 studies) | ⊕⊕⊕⊝ **moderate**^1^ |  |
| **10-Meter Walking Test (10-MWT)** |  | The mean 10-meter walking test (10MWT) in the intervention groups was **0.029 higher** (0.081 lower to 0.139 higher) |  | 144 (3 studies) | ⊕⊕⊕⊝ **moderate**^1^ |  |
| **Sensory Organization Test somatosensory (SOT somatosensory)** Dynamic balance board |  | The mean sensory organization test somatosensory (SOT somatosensory) in the intervention groups was **0.081 higher** (2.259 lower to 2.422 higher) |  | 52 (2 studies) | ⊕⊕⊝⊝ **low**^1,2^ |  |
| **Sensory Organization Test vision (SOT vision)** Dynamic balance board |  | The mean sensory organization test vision (SOT vision) in the intervention groups was **1.06 higher** (4.193 lower to 6.313 higher) |  | 52 (2 studies) | ⊕⊕⊕⊝ **moderate**^1^ |  |
| **Sensory Organization Test vestibular (SOT vestibular)** Dynamic balance board |  | The mean sensory organization test vestibular (SOT vestibular) in the intervention groups was **4.832 lower** (25.393 lower to 15.729 higher) |  | 52 (2 studies) | ⊕⊝⊝⊝ **very low**^1,2^ |  |
| **Parkinson's Disease Questionnaire - 39 total (PDQ-39 total)** Questionnaire |  | The mean parkinson's disease questionnaire - 39 total (PDQ-39 total) in the intervention groups was **1.467 lower** (7.968 lower to 5.033 higher) |  | 111 (4 studies) | ⊕⊕⊕⊝ **moderate**^1^ |  |
| **Parkinson's Disease Questionnaire - 39 mobility (PDQ-39 mobility)** Questionnaire |  | The mean parkinson's disease questionnaire - 39 mobility (PDQ-39 mobility) in the intervention groups was **1.501 higher** (5.307 lower to 8.309 higher) |  | 48 (2 studies) | ⊕⊕⊕⊝ **moderate**^1^ |  |
| **Activities-specific Balance Confidence scale (ABC)** Questionnaire |  | The mean activities-specific balance confidence scale (ABC) in the intervention groups was **1.871 higher** (5.423 lower to 9.166 higher) |  | 113 (2 studies) | ⊕⊕⊕⊝ **moderate**^1^ |  |
| *The basis for the **assumed risk** (e.g. the median control group risk across studies) is provided in footnotes. The **corresponding risk** (and its 95% confidence interval) is based on the assumed risk in the comparison group and the **relative effect** of the intervention (and its 95% CI).  **CI:** Confidence interval; | | | | | | |
| GRADE Working Group grades of evidence **High quality:** Further research is very unlikely to change our confidence in the estimate of effect.  **Moderate quality:** Further research is likely to have an important impact on our confidence in the estimate of effect and may change the estimate. **Low quality:** Further research is very likely to have an important impact on our confidence in the estimate of effect and is likely to change the estimate. **Very low quality:** We are very uncertain about the estimate. | | | | | | |
| ^1^ There were less than 400 participants in total. ^2^ I^2^ > 50% | | | | | | |
